# In Vitro Screening of *Sargassum* spp. Ethanolic Extracts for Antioxidant Activity and Inhibition of Disease-Associated Enzymatic Targets Related to Diabetes, Alzheimer’s Disease, and Parkinson’s Disease

**DOI:** 10.3390/ijms27198643

**Published:** 2026-09-27

**Authors:** Md Yousof Ali, Se Eun Park, Hyun Ah Jung, Jae Sue Choi

**Affiliations:** 1 Department of Clinical Neurosciences, Hotchkiss Brain Institute and Alberta Children’s Hospital Research Institute, Cumming School of Medicine, University of Calgary, Calgary, AB T2N 4N1, Canada; mdyousof.ali@ucalgary.ca; 2 Department of Food and Life Science, Pukyong National University, Busan 48513, Republic of Korea; 3 Department of Biomedical Science, Asan Medical Institute of Convergence Science and Technology, Seoul 05505, Republic of Korea; 4 Department of Food Science and Human Nutrition, Jeonbuk National University, Jeonju 54896, Republic of Korea

**Keywords:** *Sargassum* species, brown algae, diabetes, Alzheimer’s disease, antioxidant, health benefits

## Abstract

*Sargassum* is a large and diversified genus of marine brown algae that is distributed widely in tropical and subtropical areas. They have been used for centuries in Asian countries, and have demonstrated a variety of health advantages, including anti-cardiovascular disease, anti-osteoarthritis, anti-cancer, anti-inflammatory, antioxidant, and hypopigmentation effects. However, the putative anti-diabetic, anti-Alzheimer’s, and anti-Parkinson’s diseases activities of various *Sargassum* spp. have not been investigated and compared thoroughly. Thus, the purpose of this study was to examine the anti-diabetic, anti-Alzheimer’s, and anti-Parkinson’s diseases’ activities of ten *Sargassum* and one *myagroides* species collected off the Korean coast. In this study, we examined the inhibitory activity of 70% ethanolic extract of the *Sargassum* and *myagroides* species against protein tyrosine phosphatase 1B (PTP1B), α-glucosidase, peroxynitrite (ONOO^–^), acetylcholinesterase (AChE), butyrylcholinesterase (BChE), BACE1, MAO-A, and MAO-B. Among the *Sargassum* and *myagroides* species tested, *S. hemiphyllum*, *S. filicinum*, and *S. miyabei* showed potent PTP1B inhibitory activity (IC_50_ values of 2.11, 1.87, and 6.49 μg/mL, respectively), α-glucosidase inhibitory activity (IC_50_ values of 3.67, 2.30, and 11.12 μg/mL, respectively), and ONOO^–^ scavenging activity, with IC_50_ values of 2.07, 4.49, and 2.46 μg/mL, respectively. Moreover, *S. miyabei* exhibited the highest BACE1 and BChE inhibitory activity, with IC_50_ values of 3.38 and 39.76 μg/mL, respectively. The highest MAO-A inhibitory activity was seen in *S. miyabei, S. serratifolium*, and *S. macrocarpum*, with IC_50_ values of 16.91, 12.46, and 14.02 μg/mL, respectively. Therefore, our findings showed that *Sargassum* species have health benefits and may be useful in treating diabetes and neurological disorders.

## 1. Introduction

*Sargassum* (F. Sargassaceae), a genus of brown algae known as sea holly, belongs to the order Fucales, subclass Cyclosporeae, and class Phaeophyceae, and is one of the most complex Phaeophyceae genera [1]. Agardh identified *Sargassum* first in 1820, and according to the algal database, there are 537 species names for it, 358 of which have been accepted scientifically. *Sargassum* spp. have both bushy and linear growth patterns, with stipes that range from 1 to 20 cm in length. Its main axes are short, cylindrical, or flattened in sections, and display the remnants of deciduous branches. *Sargassum* spp. are distributed worldwide and are found primarily in the Atlantic, Pacific, and Indian Oceans, where they occupy largely tropical and subtropical environments [2]. *Sargassum* spp. have long been a part of human culture, with applications as food, animal feed, and folk medicine remedies. Since ancient times, many regions have used *Sargassum* spp. for various purposes, such as producing vegetables, fertilizer, flower inducers, insect repellents, and drinks with purported health advantages [2,3]. In addition, they have been used in traditional Chinese medicine (TCM) for approximately 2000 years to cure a number of disorders, such as thyroid, scrofula, edema, phlegm elimination, and chronic bronchitis [2,4]. *Sargassum* is used also as a food or medicine in Korea, China, and Japan to cure scrofula, edema, arteriosclerosis, and skin disorders [4,5,6,7,8].

In addition to the traditional applications stated above, *Sargassum* biomass is also considered a valuable resource with tremendous promise for healthcare and pharmaceuticals because of its high concentration of bioactive secondary compounds [1,2]. Recently, many researchers have become interested in *Sargassum* spp. because of the therapeutic potentials of pure compounds, such as phenolic compounds (phlorotannins), terpenoids, phytosterols, vitamin C, vitamin E, β-carotene, dietary fiber, and polysaccharides isolated from various *Sargassum* spp. [2,4]. These isolated compounds have a wide range of biological activities, including antiviral, analgesic, anti-microbial, cytotoxic, anti-tumor, fibrinolytic, antipyretic, anti-inflammatory, cardioprotective, hepatoprotective, antioxidant, immune-modulatory, anti-coagulant, anti-cancer, anti-diabetic, and anti-neurodegenerative disorders [2,4,9,10].

Neurodegenerative diseases are a group of diseases characterized by the slow and irreversible loss of neurons, neurotransmitters, and neuronal structures in specific areas of the brain [11]. These diseases include Alzheimer’s disease (AD), in which the loss of hippocampal and cortical neurons and neuronal structures causes memory loss and cognitive deficiency, amyotrophic lateral sclerosis, in which abnormal proteins accumulate in brain tissues and cause muscular weakness [12], and Parkinson’s disease (PD), in which the loss of neurons and neuronal structures of the basal ganglia leads to anomalies in the coordination of movement [11,13]. These diseases are quite common today and pose a significant medical and public health problem in all human communities. One of the current therapeutic applications for neurodegenerative diseases is the reversal of neuron, neurochemicals, and neuronal structures that are lost throughout the disease process.

Two main important enzymes, acetylcholinesterase (AChE) and butyrylcholinesterase (BChE) [14], are considered first-line treatments for AD, but BACE1 (β-site amyloid precursor protein cleavage enzyme), which is also responsible for amyloid beta (Aβ) buildup, has also been linked to the development of AD [15]. As a result, it is plausible that inhibiting these enzymes’ activities may reverse neuron, neurochemical, and neuronal designs, and thus offer an expected therapeutic target for the treatment of AD. Another enzyme group known as monoamine oxidases (MAOs) is responsible for PD. As a result, suppressing MAOs, which catalyze the oxidative deamination of dopamine and other biogenic amines, is one technique used to maintain dopamine levels in the substantia nigra pars compacta [16]. Therefore, inhibiting MAOs may increase dopamine levels in the striatum and alleviate some of the symptoms associated with PD and other neurodegenerative disorders [17,18].

Diabetes, a chronic metabolic disease, poses serious threats to human health and adversely affects millions of people’s quality of life [19]. The past two decades have witnessed an alarming increase in the number of diabetic patients around the globe, particularly those with type 2 diabetes mellitus (T2DM) [20]. The deficiency or failure of insulin secretion, or both, leads to high blood sugar levels, and thus indicates T2DM [20,21]. At present, T2DM is ranked as one of the major health concerns largely because of its continued increased pervasiveness throughout the world, and because it is responsible for the high morbidity and mortality rates related to chronic diabetic complications [21,22]. Currently, the proposed therapeutic approaches to overcome this disorder include controlling the digestion and absorption of carbohydrates via α-glucosidase inhibitors [23], and modulating insulin receptor phosphorylation through protein tyrosine phosphatase 1B (PTP1B) inhibitors together with insulin therapy [22,23].

Approximately 30 distinct species of *Sargassum* have been discovered in the area of Jeju Island, Busan, and Geoje on the Korean coast. Previously, it was reported that 11 species of Korean *Sargassum* contain significant amounts of total phenolic and total flavonoids, and have high antioxidant and anti-tyrosinase effects [6,24]. However, to the best of our knowledge, there has been no comprehensive analysis and comparison of various *Sargassum* species’ biological activity. Here, the Marine Bio-Resource Information System (MBRIS) collected 10 species off the Korean coast—*S. hemiphyllum*, *S. coreanum*, *S. confusum*, *S. siliquastrum*, *S. miyabei*, *S. serratifolium*, *S. filicinum*, *S. micracanthum*, *S. macrocarpum*, *S. yendoi*—and one *myagroides* species, *M. myagroides*, and we analyzed and compared their anti-AD, anti-PD, anti-diabetic, and antioxidant activities. The goal of this study was to investigate these *Sargassum* and *myagroides* species’ bioactivities and their potential ability to prevent neurodegenerative disorders and diabetes.

## 2. Results and Discussion

### 2.1. PTP1B Inhibitory Activity of the Sargassum Species Selected

PTP1B is a member of the PTP superfamily that is found on the cytoplasmic surface of the endoplasmic reticulum and is expressed in a variety of tissues, including liver, muscle, and fat [25,26]. PTP1B has been implicated as a key negative regulator of the insulin and leptin signaling pathways [27]. It plays a crucial role in phosphorylating and inactivating the insulin receptor to shut down insulin signaling. Further, insulin receptors and insulin-receptor substrate-1 interact directly to hydrolyze phosphorylated tyrosine, which leads to impaired glucose uptake [28]. For example, PTP1B knockdown mice exhibited improved insulin sensitivity in glucose and insulin tolerance testing, which suggests that PTP1B largely modulates insulin sensitivity [29,30]. As a result, PTP1B has become an active therapeutic target for the treatment of T2DM.

In this study, the different *Sargassum* and *myagroides* species extracts’ potential to inhibit PTP1B was investigated, as shown in Table 1. As Figure 1 shows, *S. filicinum*, *S. siliquastrum*, and *S. hemiphyllum* showed the highest concentration-dependent PTP1B inhibitory activity. The 70% EtOH extracts of all of the *Sargassum* and *myagroides* species selected showed strong PTP1B inhibitory activities. Among all of the species selected, *S. filicinum*, *S. siliquastrum*, *S. miyabei*, *S. yendoi*, *S. confusum*, *M. myagroides*, *S. micracanthum*, *S. hemiphyllum*, *S. macrocarpum*, *S. coreanum*, and *S. serratifolium* displayed promising PTP1B inhibitory activities, with IC_50_ values of 1.87 ± 0.09, 5.71 ± 0.25, 6.49 ± 1.09, 10.88 ± 1.53, 11.88 ± 0.81, 6.73 ± 0.84, 40.38 ± 2.25, 2.11 ± 0.07, 11.17 ± 0.45, 15.39 ± 0.39, and 10.58 ± 0.67 µg/mL, respectively. The positive control, ursolic acid, showed inhibitory activity with an IC_50_ value of 7.92 ± 0.20 µg/mL. Previously, we reported that *S. serratifolium* extract and bioactive constituents showed potent PTP1B inhibitory activity [31]. Further, several studies have indicated that various *Sargassum* species and their major constituents demonstrated anti-diabetic activity [3,6,32,33]; however, this is the first time that we evaluated and compared the anti-diabetic potential of the *Sargassum* species by inhibiting PTP1B.

### 2.2. α-Glucosidase Inhibitory Activity of the Sargassum Species Selected

α-Glucosidase is a glycoside hydrolase enzyme that is located in the brush-border surface membrane of intestinal cells. During carbohydrates’ digestion process, *α*-glucosidase cleaves the *α*-glucopyranoside link in oligosaccharides to monosaccharide [34]. Further, the production of these absorbable monosaccharides results in postprandial hyperglycemia in patients with diabetes. Therefore, inhibiting the digestion and absorption of carbohydrates by using *α*-glucosidase inhibitors slows carbohydrate digestion, stabilizes the blood glucose level, and consequently prevents hyperglycemia in diabetic patients [35]. Today, acarbose, miglitol, and voglibose, all of which have carbohydrate mimetic properties, are among the various α-glucosidase inhibitors that are used in therapeutic settings to treat type 2 diabetes [36,37,38]. However, because of their side effects, including flatulence, diarrhea, stomachache, and liver damage, it is necessary to develop natural, edible, new, efficient, and benign α-glucosidase inhibitors to treat diabetes mellitus (DM). Therefore, in this work, we tested edible *Sargassum* and *myagroides* species for their capacity to inhibit α-glucosidase. As shown in Figure 2, *S. filicinum*, *M. myagroides*, and *S. hemiphyllum* showed the highest concentration-dependent *α*-glucosidase inhibitory activity, while as Table 1 shows, most of the *Sargassum* and *myagroides* species showed significant α-glucosidase inhibitory activities. Among the tested species, *S. filicinum*, *S. siliquastrum*, *S. miyabei*, *S. yendoi*, *S. confusum*, *M. myagroides*, *S. hemiphyllum*, and *S. macrocarpum* were found to be the most potent α-glucosidase inhibitors with IC_50_ values of 2.30 ± 0.06, 51.52 ± 2.69, 12.12 ± 0.46, 14.82 ± 0.03, 24.71 ± 1.28, 11.17 ± 0.51, 3.67 ± 0.17, and 61.72 ± 3.21 µg/mL, respectively, all of which were significantly higher than the positive control, acarbose (IC_50_ = 92.97 ± 3.81 µg/mL). Furthermore, *S. micracanthum*, *S. coreanum*, and *S. serratifolium* were inactive in the α-glucosidase inhibitory assay at the tested concentration of 100 µg/mL. Few *Sargassum* species have previously been reported to have α-glucosidase inhibitory activity [6,31,39,40], which is compatible with our findings; nevertheless, in this study, we evaluated and compared the anti-diabetic potential of the *Sargassum* species by inhibiting the α-glucosidase.

### 2.3. ONOO^–^ Scavenging Activity of the Sargassum Species Selected

Nitric oxide (NO) is a free radical generated in the body that may interact with superoxide (O_2_^−^) to form peroxynitrite (ONOO^–^), which is a potent oxidant that causes a variety of forms of oxidative damage [41,42]. ONOO^–^ is also responsible for the process of protein oxidation, nitration, lipid peroxidation, thiol oxidation, amino acid modification, and DNA damage [42,43]. Moreover, ONOO^–^ toxicity has been linked to a number of disorders, including cancer and cardiovascular and neurological diseases [42,43,44,45]. A number of compounds, including glutathione, methionine, cysteine, melatonin, and serotonin, are now thought to neutralize cytotoxic ONOO^–^. Because of the obvious cytotoxicity and tissue destruction that ONOO^–^ causes, there has been considerable interest in natural antioxidants’ capacity to reduce ONOO^–^-induced damage. As a result, research on ONOO^–^ scavengers and their response mechanisms may be a useful tool to develop preventative therapy measures. As a result, as shown in Table 1, we evaluated the ONOO^–^ scavenging activity of the edible *Sargassum* and *myagroides* species. As illustrated in Figure 3, *S. filicinum*, *S. miyabei*, and *S. hemiphyllum* showed the highest concentration-dependent ONOO^–^ scavenging activity. The 70% EtOH extracts of all of the *Sargassum* and *myagroides* species selected showed strong ONOO^–^ scavenging activity. All of the ten *Sargassum* species selected, and the one selected *myagroides* species, *S. filicinum*, *S. siliquastrum*, *S. miyabei*, *S. yendoi*, *S. confusum*, *M. myagroides*, *S. micracanthum*, *S. hemiphyllum*, *S. macrocarpum*, *S. coreanum*, and *S. serratifolium*, displayed strong ONOO^–^ scavenging activities, with IC_50_ values of 4.49 ± 0.35, 11.96 ± 0.97, 2.46 ± 0.38, 6.33 ± 0.80, 15.61 ± 0.70, 11.28 ± 2.83, 33.19 ± 2.89, 2.07 ± 0.06, 17.25 ± 0.86, 51.50 ± 0.35, and 14.30 ± 0.63 µg/mL, respectively. The inhibitory activity of the positive control, penicillamine, was shown with an IC_50_ value of 0.22 ± 0.10 µg/mL. To the best of our knowledge, this is the first time that *Sargassum* species’ ability to antioxidize by scavenging ONOO^–^ has been assessed and compared.

### 2.4. AChE, BChE, and BACE1 Inhibitory Activities of the Sargassum Species Selected

Alzheimer’s disease (AD) is a progressive neurologic disorder that affects people primarily in their later years [14]. The lack of the neurotransmitter acetylcholine (ACh) is one of the factors that contributes to AD’s pathophysiology. The regulation of ACh in the brain is carried out by two cholinesterase enzymes: butyrylcholinesterase (BChE) and acetylcholinesterase (AChE). Both enzymes break down ACh into acetate and choline [46]. In the healthy brain, AChE rather than BChE regulates ACh. However, the BChE level rises from 0.6 in the healthy brain to as high as 11 in the cortical regions of a patient affected by AD [47,48]. Therefore, the BChE enzyme’s activity is also necessary to control ACh. As a result, inhibition of cholinesterase enzymes has emerged as one of the potential therapies for AD. Treatment with cholinesterase inhibitors has been found to improve AD patients’ cognitive function [15,49,50]. The ten *Sargassum* and one *myagroides* species’ potential to inhibit AChE and BChE were evaluated, and the results are presented in Table 2. None of the species showed AChE inhibitory activity at the tested concentration of 100 µg/mL. On the other hand, *S. miyabei*, *S. macrocarpum*, and *S. filicinum* showed significant BChE inhibitory activity, with IC_50_ values of 39.76 ± 5.29, 77.98 ± 3.69, and 93.96 ± 4.51 µg/mL, respectively. As shown in Figure 4, *S. miyabei*, *S. macrocarpum*, and *S. filicinum* showed the most potent concentration-dependent BChE inhibitory activity, whereas *S. serratifolium*, *S. siliquastrum*, *S. confusum*, *S. hemiphyllum*, *S*. *micracanthum*, *S. coreanum*, *S*. *yendoi*, and *M. myagroides* were inactive in the BChE inhibitory assay at the tested concentration of 100 µg/mL. The inhibitory activity of the positive control, berberine, was shown with IC_50_ values of 0.34 ± 0.02 and 11.49 ± 0.90 µg/mL, which were used in each AChE and BChE assay.

Further, β-secretase/BACE1, a membrane-bound aspartyl-protease enzyme, has also been related to the catalytic processing of the amyloid precursor protein (APP) to generate amyloidogenic plaques known as amyloid beta (Aβ) [15]. BACE1 is expressed primarily in neurons, astrocytes, and dendrocytes in the brain [51]. In general, the primary pathogenic event in AD is the overproduction and/or decreased clearance of Aβ peptides. Further, Aβ overproduction initiates aggregation to generate neurotoxic Aβ plaques. As a result, Aβ accumulation is hypothesized to be an early, perhaps even initating, event in the pathogenesis of AD-related diseases such as dementia and cell death [15,52]. In addition, it has been reported that BACE1 activity in mouse, monkey, and human brains increases with age, which is the primary risk factor for AD [53]. Therefore, it is conceivable that the inhibition of BACE1 activity provides a likely therapeutic target for the treatment of AD. Accordingly, in this study, we evaluated and compared the ten different *Sargassum* and one *myagroides* species for their capacity to inhibit BACE1. As shown in Table 2, all of the species showed promising BACE1 inhibitory activities. As illustrated in Figure 5, *S. miyabei*, *S. filicinum*, and *S. coreanum* showed the most potent concentration-dependent BACE1 inhibitory activity. All 11 of the species selected, *S. filicinum*, *S. siliquastrum*, *S. miyabei*, *S. yendoi*, *S. confusum*, *M. myagroides*, *S. micracanthum*, *S. hemiphyllum*, *S. macrocarpum*, *S. coreanum*, and *S. serratifolium*, displayed promising BACE1 inhibitory activities with IC_50_ values of 8.76 ± 0.72, 10.70 ± 1.51, 3.88 ± 0.75, 17.71 ± 2.00, 13.37 ± 2.80, 9.74 ± 1.95, 63.50 ± 1.12, 16.88 ± 0.49, 13.14 ± 2.68, 7.14 ± 0.51, 11.55 ± 1.49 µg/mL, respectively. The inhibitory activity of the positive control, quercetin, was shown with an IC_50_ value of 3.5 ± 0.24 µg/mL. Previously, we also reported that *S. serratifolium* extract and its bioactive constituents showed significant BACE1 inhibitory activity [54]. Moreover, it has been reported that various marine-derived compounds inhibit BACE1 [55]. However, this is the first evaluation and comparison of the anti-AD potential of *Sargassum* species by inhibiting BACE1.

### 2.5. The 11 Sargassum Species’ Ability to Inhibit Monoamine Oxidases (MAOs)

Monoamine oxidases (MAOs) are flavoenzymes that catalyze the oxidation of biogenic and xenobiotic amines, including dopamine, serotonin, norepinephrine, and adrenaline [56]. Thus, MAOs play an important role in controlling monoamine levels in the central nervous system (CNS). There are two isoenzyme types of MAO in the human brain: MAO-A and MAO-B. MAO-A is a therapeutic target, with its inhibitors frequently prescribed for anxiety disorders such as depression, while MAO-B is used to treat such neurological disorders as PD [57]. As a result, these enzymes’ inhibition has been one of the effective strategies used to discover antidepressant and neuroprotective molecules. Thus, the *Sargassum* and *myagroides* species selected were tested for their ability to inhibit MAO-A and MAO-B. The results are shown in Table 2. None of the species showed MAO-B inhibitory activity at the tested concentration of 100 µg/mL. On the other hand, *S. serratifolium*, *S. macrocarpum*, *S. miyabei*, and *S. filicinum* showed significant inhibition of MAO-A, with IC_50_ values of 12.46 ± 0.73, 14.02 ± 0.22, 16.91 ± 0.87, and 91.32 ± 3.99 µg/mL, respectively. As shown in Figure 6, *S. serratifolium*, *S. miyabei*, and *S*. *macrocarpum* showed the most promising concentration-dependent MAO-A inhibitory activity. Furthermore, *S. coreanum*, *S. confusum*, *S. siliquastrum*, *S. micracanthum*, *S*. *hemiphyllum*, *S. yendoi*, and *M. myagroides* were inactive in the MAO-A inhibitory assay at the tested concentration of 100 µg/mL. The inhibitory activity of the positive control, Deprenyl HCl, was shown with IC_50_ values of 2.47 ± 0.11, and 0.31 ± 0.01 µg/mL, which were used in MAO-A and MAO-B assays. We found some extracts generated negative inhibition values. This occurs when the measured assay signal of the sample exceeds that of the enzyme control, resulting in a calculated inhibition percentage below zero. Such values do not indicate inhibitory activity and should not be interpreted as biologically relevant enzyme activation. Because the assays were performed using crude *Sargassum* and *myagroides* extracts, matrix components such as pigments and other secondary metabolites may contribute to signal interference and produce apparent negative inhibition values. Therefore, negative values were interpreted as a lack of inhibitory activity under the tested conditions. Previously, our lab reported that the brown algae *S. macrocarpum* and *Eisenia bicyclis*, as well as their major constituents, exhibited inhibitory activities against both MAO-A and MAO-B [58,59]. However, this is the first evaluation and comparison of the 11 *Sargassum* species’ anti-PD potential by inhibiting MAO-A and MAO-B.

The most active *Sargassum* species extracts—*S. hemiphyllum*, *S. siliquastrum*, *S. filicinum*, *S. serratifolium*, *S. miyabei*, and *S. yendoi*—as well as their main constituents, exhibited a wide range of biological activity. For example, different extracts and fractions of *S. siliquastrum* have been shown to have pharmacological activities, such as anti-AD, anti-inflammatory, anti-diabetic, antioxidant, anti-obesity, and anti-tyrosinase effects [4,6,10,24]. Further, *S. siliquastrum* is high in meroterpenoids, farnesylacetones, mojabanchromanol, tetraprenyl hydroquinone nahocol, and isonahocol, all of which have been attributed to have a variety of pharmacological activities, such as anti-AD, anti-cancer, neuroprotective, anti-diabetic, and anti-inflammatory activities [2,4,5,31]. A further investigation reported that the most active *S. filicinum* ethanol extract demonstrated anti-inflammatory [2,4,6], anti-diabetic [2,6], antioxidant [4,6,10,24], and anti-tyrosinase [24] activities. *S. filicinum* contains various bioactive compounds, such as fucoxanthin, phenolics, polysaccharides, sterols, Apo-9 fucoxanthinone, octatriene, mojabanchromanol, (−)-loliolide, sargachromanol E, and sargachromenol [2,10], which also have antioxidant and anti-inflammatory properties [2,4,10].

*S. serratifolium* is another important *Sargassum* spp. with diverse pharmacological properties. We reported previously that *S. serratifolium* extract and different fractions showed anti-AD activity by inhibiting AChE, BChE, and BACE1 [54], and anti-diabetic activity by inhibiting PTP1B and α-glucosidase enzymes [31]. In addition, *S. serratifolium* extracts and fractions demonstrated antioxidant [6,10], hepatoprotective [10], anti-inflammatory [60], anti-obesity [61], anti-microbial [62], and anti-tyrosinase [24] properties. *S. serratifolium* contains a variety of compounds, including sargahydroquinoic acid, sargachromanol, sargahydroquinoic acid, and sargaquinoic acid [10,31,54], all of which have anti-diabetic and anti-AD properties [31,54]. Further, numerous pharmacological effects, including anti-diabetic [63], antioxidant, anti-tyrosinase [4,10,24], anti-inflammatory, and anti-obesity [6,64] properties, have been found for *S. hemiphyllum* extracts and fractions. Several types of bioactive compounds, such as phenolics, flavonoids, sulfated polysaccharides, and crude phlorotannins, are present in *S. hemiphyllum* [4,6,10,24].

Similarly, among the different *Sargassum* spp. reported, *S. miyabei* has the highest total phenolic and flavonoid content [24]. *S. miyabei* 70% ethanolic extract displayed potent antioxidant activity by scavenging ABTS, superoxide, DPPH, and FRAP, as well as anti-tyrosinase activity by inhibiting L-tyrosine and L-DOPA [6,10,24]. The ethanolic extract of *S. miyabei* also showed anti-inflammatory [65], antioxidant [24], and anti-adipogenic properties [66]. Sargahydroquinoic acid and sargachromanol, two major compounds, have been detected in the *S. miyabei* extract. Moreover, *S. yendoi* extract also exhibited antioxidant activity by scavenging ABTS, superoxide, DPPH, and FRAP, as well as anti-tyrosinase activity by inhibiting L-tyrosine and L-DOPA [6,24]. The most active *Sargassum* species major compounds are shown in Figure 7.

## 3. Materials and Methods

### 3.1. Plant Materials and Sample Preparation

*Sargassum hemiphyllum* (TURNER) C. AGARDH 1820 (MRS00200092016), *S. confusum* C. AGARDH 1824 (MRS00200092011), *S. filicinum* HARVEY 1860 (MRS00200092015), *S. micracanthum* (Kützing) Endlicher 1843 (MRS00200092012), and *S. coreanum* J. Agardh 1889 (MRS00200092010) were collected along the coast of Jeju Island, South Korea in 2011. *S. miyabei* Yendo 1907 (MRS00200092031) was collected along the coast of Ulleung Island, South Korea in 2015. *S. macrocarpum* C. AGARDH 1820 (MRS00200092094), *Myagropsis myagroides* (Mertens ex Turner) Fensholt 1955 (MRS00200092025), *S. siliquastrum* (MERTENS ex TURNER) C. AGARDH 1820 (MRS00200092096), *S. serratifolium* (C. Agardh) C. Agardh 1820 (MRS00200092098), and *S. yendoi* Okamura & Yamada in Yamada 1938 (MRS00200092100) were collected along the coast of Jeju Island in 2016. *Myagropsis myagroides* is a member of the family Sargassaceae and is taxonomically classified under the genus *Myagropsis* and species *myagroides*. The specimens’ identity was confirmed by an algal biologist (Nam Gil Kim) at the Department of Marine Biology and Aquaculture, Gyeongsang National University, South Korea. Ten standardized *Sargassum* extract powders (dried *Sargassum* samples were extracted with 70% ethanol through ultra-sonication) were obtained officially from the MBRIS.

### 3.2. Sargassum and Myagroides Species Collection, Extraction, Yields, and Standardization

All seaweed samples were collected from Jeju Island and Ulleung Island, South Korea during the collection periods described above. Following collection, the samples were thoroughly washed with seawater and tap water to remove salts, sand, and epiphytes, and the leafy thalli were subsequently shade dried. Dried seaweed materials (10 g each) were extracted with 70% ethanol using ultrasonic-assisted extraction and then lyophilized. The yields of the 70% ethanolic extracts were as follows: *S. hemiphyllum* (250 mg), *S. confusum* (125 mg), *S. filicinum* (75 mg), *S. micracanthum* (855 mg), *S. coreanum* (780 mg), *S. miyabei* (135 mg), *S. macrocarpum* (680 mg), *M. myagroides* (75 mg), *S. siliquastrum* (860 mg), *S. serratifolium* (450 mg), and *S. yendoi* (30 mg). Voucher specimens of all collected samples have been deposited in the laboratory of J. S. Choi for future reference. Extraction with 70% ethanol is not a legally mandated method for botanical materials; however, it is widely used in phytochemical and pharmacological research because of its ability to efficiently extract both hydrophilic and moderately lipophilic constituents. In the present study, dried seaweed samples were extracted using 70% (*v*/*v*) ethanol at a defined solid-to-solvent ratio, followed by filtration and concentration under reduced pressure. The USP <565> chapter on botanical extracts recognizes water, alcohols, hydroalcoholic mixtures, and other suitable solvents as acceptable extraction media for botanical materials. Furthermore, in Korea, standards and testing methods for herbal medicines are regulated by the Ministry of Food and Drug Safety (MFDS) through the Korean Pharmacopoeia (KP) and the Korean Herbal Pharmacopoeia (KHP), which provide general requirements, quality standards, and analytical procedures for herbal medicinal products.

### 3.3. Chemicals and Reagents

Yeast α-glucosidase, *p*-nitrophenyl phosphate (*p*NPP), *p*-nitrophenyl α-D-glucopyranoside (*p*NPG), acarbose (>95% purity), ursolic acid (> 90% purity), berberine (>99%), dimethyl sulfoxide (DMSO), quercetin (>95%), L-2-amino-3-mercapto-3-methylbutanoic acid (L-penicillamine), electric eel acetylcholinesterase (AChE, EC3.1.1.7), horse serum butyrylcholinesterase (BChE, EC 3.1.1.8), acetylcholine chloride (ACh), butyryl thiocholine chloride (BCh), and 5,5′-dithiobis [2-nitrobenzoic acid] (DTNB) were purchased from Sigma-Aldrich Co. (St Louis, MO, USA). PTP1B (human recombinant) was purchased from Biomol^®^ International LP (Plymouth Meeting, PA, USA). The BACE1 (β-secretase) FRET assay kit was purchased from Pan Vera Co. (Madison, WI, USA). Recombinant hMAO-A and hMAO-B, L-deprenyl·HCl, and clorgyline·HCl, 3-isobutyl-1-methylxanthine (IBMX) were purchased from Sigma Aldrich (Darmstadt, Germany). ONOO^–^ was purchased from Cayman (Ann Arbor, MI, USA). High-quality dihydrorhodamine 123 (DHR 123) was purchased from Molecular Probes (Eugene, OR, USA). All other chemicals and solvents used were purchased from E. Merck, Fluka, or Sigma-Aldrich, unless otherwise stated.

### 3.4. Protein Tyrosine Phosphatase 1B (PTP1B) Inhibition Assay

The PTP1B inhibitory activities of different *Sargassum* and *myagroides* species were evaluated using *p*NPP [67]. Briefly, in each well of a 96-well plate, 40 μL of PTP1B enzyme [0.5 units diluted with PTP1B reaction buffer containing 50 mM citrate (pH 6.0), 0.1 M NaCl, 1 mM EDTA, and 1 mM DTT] was added with or without sample dissolved in 10% DMSO. Final concentration of DMSO was 0.1% and buffer containing 0.1% DMSO was used as a vehicle control. The plate was pre-incubated at 37 °C for 10 min, and then 50 μL of 2 mM pNPP in PTP1B reaction buffer was added. Following incubation at 37 °C for 20 min, the reaction was terminated by addition of 10 M NaOH. The amount of *p*-nitrophenyl produced after enzymatic dephosphorylation of *p*NPP was estimated by measuring absorbance at 405 nm using a microplate spectrophotometer (Molecular Devices, Sunnyvale, CA, USA).

### 3.5. α-Glucosidase Inhibitory Assay

The α-glucosidase inhibitory activities of different *Sargassum* and *myagroides* species were evaluated using the procedure reported by [67]. A total of 60 µL of reaction mixture containing 20 µL of 100 mM phosphate buffer (pH 6.8), 20 µL of 2.5 mM pNPG, and 20 µL of the sample dissolved in 10% DMSO was added to each well, followed by 20 µL of α-glucosidase [0.2 U/mL in 10 mM phosphate buffer (pH 6.8)]. The plate was incubated at 37 °C for 15 min, and 80 µL of 0.2 M sodium carbonate solution was then added to stop the reaction. Immediately thereafter, the absorbance was recorded at 405 nm using a microplate spectrophotometer (Molecular Devices, Sunnyvale, CA, USA). Acarbose was used as a positive control.

### 3.6. ONOO^–^ Scavenging Activity Assay

ONOO^–^ scavenging activity was assessed by a reported method [67] that monitors the production of fluorescent rhodamine 123 from non-fluorescent DHR123 in the presence of ONOO^–^. The fluorescence intensity of oxidized DHR123 was evaluated using a microplate fluorescence reader (Bio-Tek Instruments Inc., FLx800, Winooski, UT, USA) at excitation and emission wavelengths of 480 and 530 nm, respectively. L-Penicillamine was used as a positive control.

### 3.7. In Vitro BACE1 Enzyme Assay

The in vitro BACE1 enzyme assay was carried out according to the manual provided, with slight modifications as described in a previous study [68]. Briefly, a mixture of 10 μL of assay buffer (50 mM sodium acetate, pH 4.5), 10 μL of BACE1 (1.0 U/mL), 10 μL of the substrate (750 nM Rh-EVNLDAEFK-Quencher in 50 mM, ammonium bicarbonate) and 10 mL of the test samples was dissolved in 10% DMSO and incubated for 60 min at 25 °C in the dark. The proteolysis of two fluorophores (Rh-EVNLDAEFK-Quencher) by BACE1 was determined by monitoring formation of the fluorescent donor (Rh-EVNL) at wave lengths of 530–545 nm for excitation and 570–590 nm for emission. Fluorescence was measured with a microplate spectrofluorometer (Gemini EM, Molecular Devices, Sunnyvale, CA, USA). Specifically, each reaction was excited at 545 nm and the emission intensity was recorded at 585 nm. Quercetin was the positive control.

### 3.8. In Vitro Cholinesterase Enzyme Assay

The inhibitory activities of different *Sargassum* and *myagroides* species against cholinesterases were measured using the spectrophotometric method [68], developed with ACh and BCh as the substrates to assay inhibition of AChE and BChE, respectively. The reaction mixture contained 140 μL of sodium phosphate buffer (pH 8.0), 20 μL of test sample solution (final concentration of the *Sargassum* spp. 100 μg/mL), and 20 μL of either AChE or BChE solution, which were mixed and incubated for 15 min at room temperature. All test samples and the positive control (berberine) were dissolved in 10% analytical grade DMSO. The reactions were initiated upon addition of 10 μL of DTNB and 10 μL of either ACh or BCh, and their hydrolysis was monitored by tracking the formation of the yellow 5-thio-2-nitrobenzoate anion that results from the reaction of DTNB with thiocholine, and the enzyme released, in triplicate samples in 96-well microplates, using a microplate spectrophotometer (Molecular Devices, Sunnyvale, CA, USA) at 412 nm for 15 min. The percent inhibition was calculated as (1 − S/E) × 100, where S and E are the presence or absence of samples and the enzyme activities, respectively.

### 3.9. Human Monoamine Oxidase Inhibition Assay

The inhibitory activities on *h*MAO-A and *h*MAO-B of different *Sargassum* and *myagroides* species were evaluated using a MAO-GloTM chemiluminescent assay kit (Promega, Madison, WI, USA). The experimental procedures for this experiment were described earlier [69]. Briefly, 12.5 µL of the test *Sargassum* extracts or l-deprenyl/clorgyline HCl was added to a 12.5 µL aliquot of beetle luciferin derivative substrate (the initial concentrations of hMAO-A and hMAO-B were 0.08–5.59 µg/mL) in each well of a 96-well plate. An enzyme solution (25 µL) was then added to the test samples to initiate the reaction. After an hour of incubation at 25 °C, a reconstituted luciferin detection reagent (50 µL) was added to each well to stop the reaction and produce a luminescent signal. The final mixture was incubated for an additional 20 min at 25 °C. Then, the luminescence was recorded on a FilterMax F5 Multi-Mode microplate reader (Molecular Devices LLC, San Jose, CA, USA). The amount of luminescence was directly proportional to the residual activity of the hMAO isoenzymes.

### 3.10. Statistical Analyses

All results are expressed as the means ± SD of triplicate samples. Results were analyzed using one-way ANOVA and Student’s *t*-test where appropriate (Systat Inc., Evanston, IL, USA). A *p* value < 0.05 was considered statistically significant (* *p* < 0.05, ** *p* < 0.001, and *** *p* < 0.0001).

## 4. Conclusions

In summary, East Asian countries have traditionally used *Sargassum* spp. as a dietary supplement and functional food. The 70% EtOH extract of *Sargassum* spp. was used for this study to examine potential anti-diabetic, antioxidant, anti-AD, and anti-PD activities. Remarkable inhibitory activities against PTP1B, α-glucosidase, ONOO^–^, and BACE1 were demonstrated by the *Sargassum* extract. In this study, we investigated and compared ten *Sargassum* and one *myagroides* species for their ability to inhibit several enzymes that are associated with human diseases. *Sargassum*’s beneficial effects are attributable largely to bioactive compounds, such as terpenoids, sterols, polysaccharides, polyphenols, and pheophytin. Various *Sargassum* species from Korea have been explored for their antioxidant, anti-inflammatory, anti-diabetic, anti-obesity, and anti-cancer properties; however, the majority of the species are yet to be tested for diabetes and neurological disorders. However, the present results are based solely on enzyme-based and chemical assays and should not be interpreted as evidence of therapeutic efficacy. Further studies involving bioactive compound isolation, mechanistic investigations, and in vivo validation are required to determine their pharmacological potential.

## Figures and Tables

**Figure 1:**
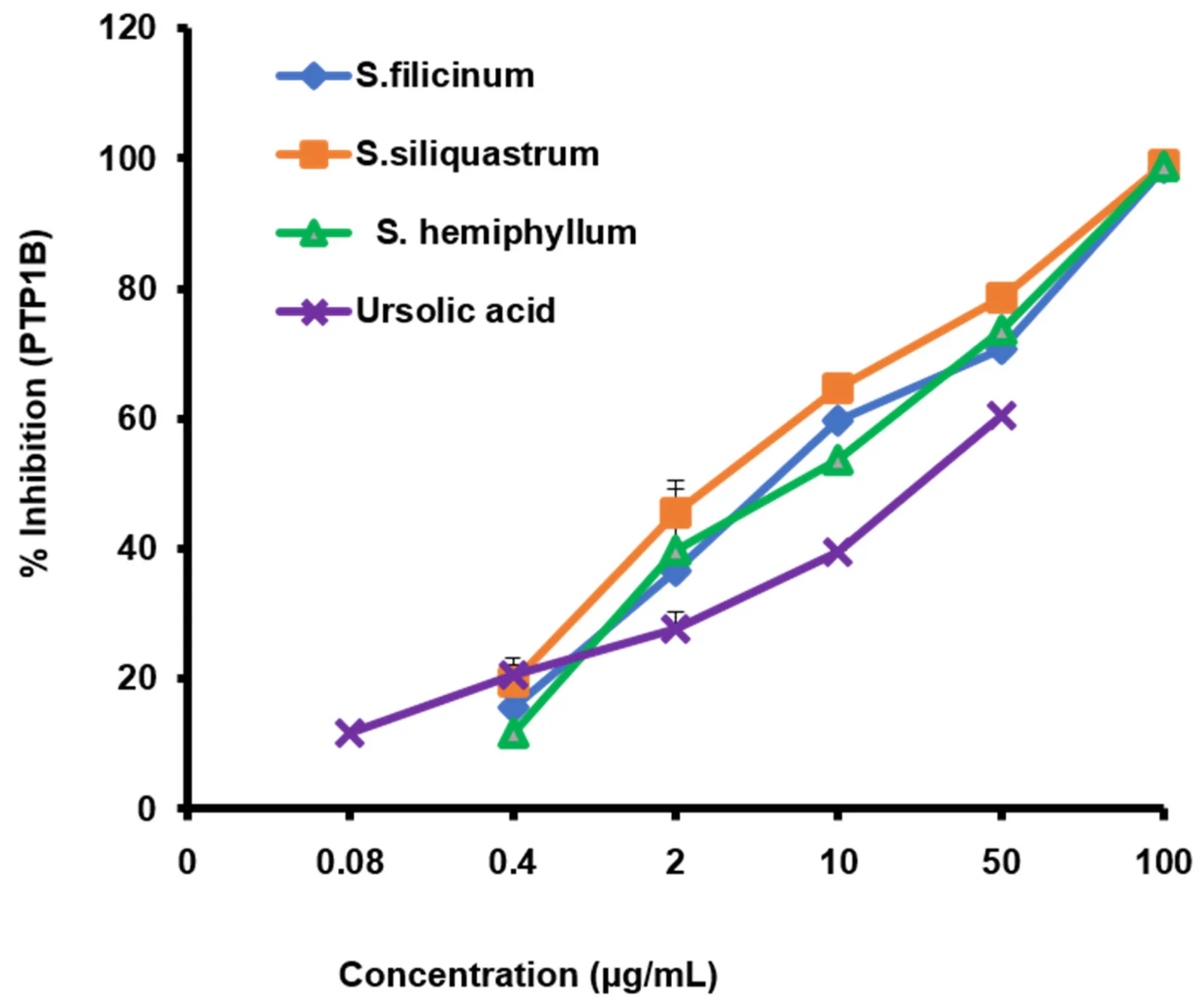
Inhibitory activity of protein tyrosine phosphatase 1B by 70% EtOH *Sargassum* spp. extract. Ursolic acid was used as a positive control. All values are expressed as the mean ± SD of triplicate experiments (*n* = 3).

**Figure 2:**
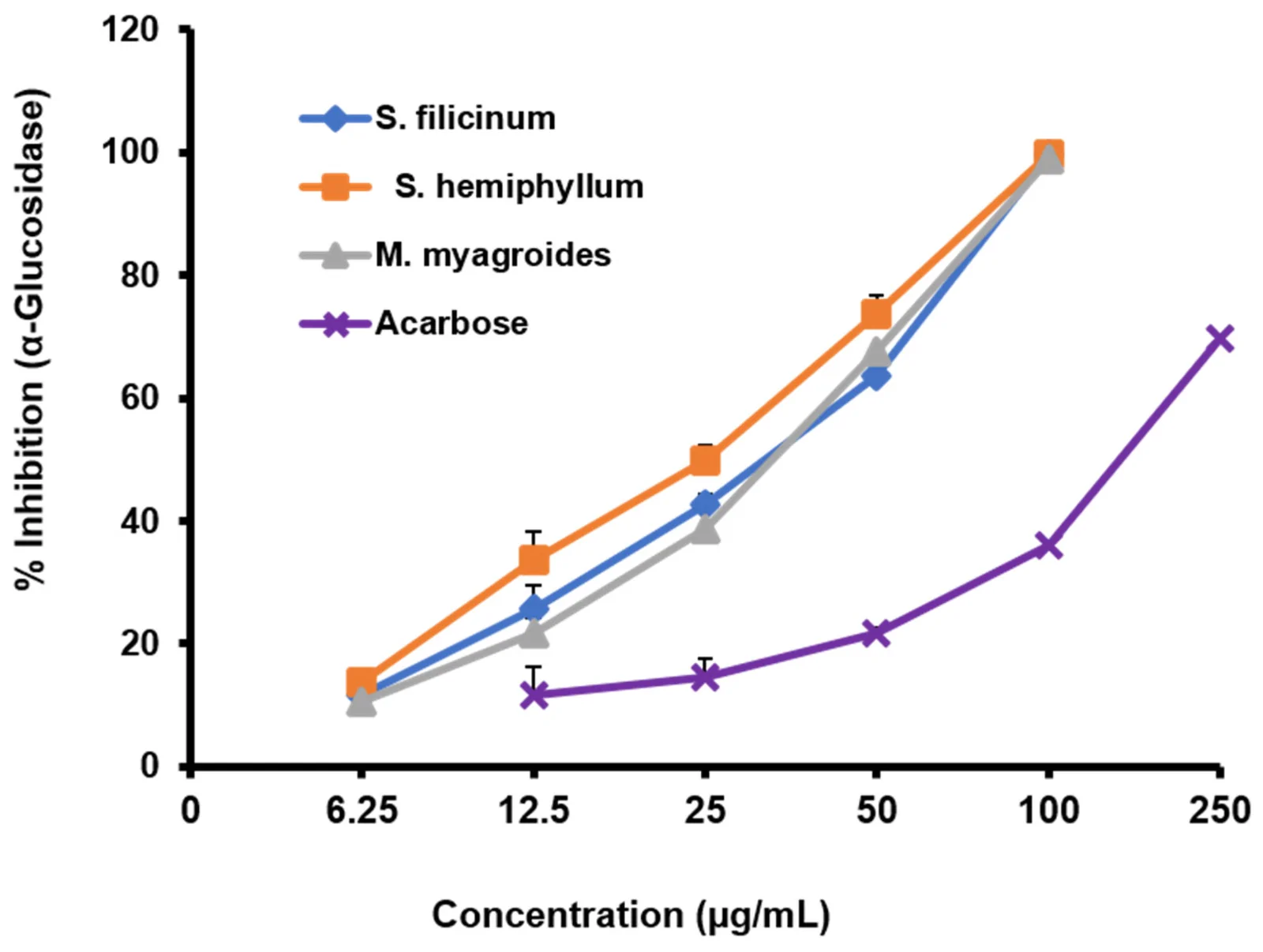
Inhibitory activity of α-glucosidase by 70% EtOH *Sargassum* and *myagroides* species extract. Acarbose was used as a positive control. All values are expressed as the mean ± SD of triplicate experiments (*n* = 3).

**Figure 3:**
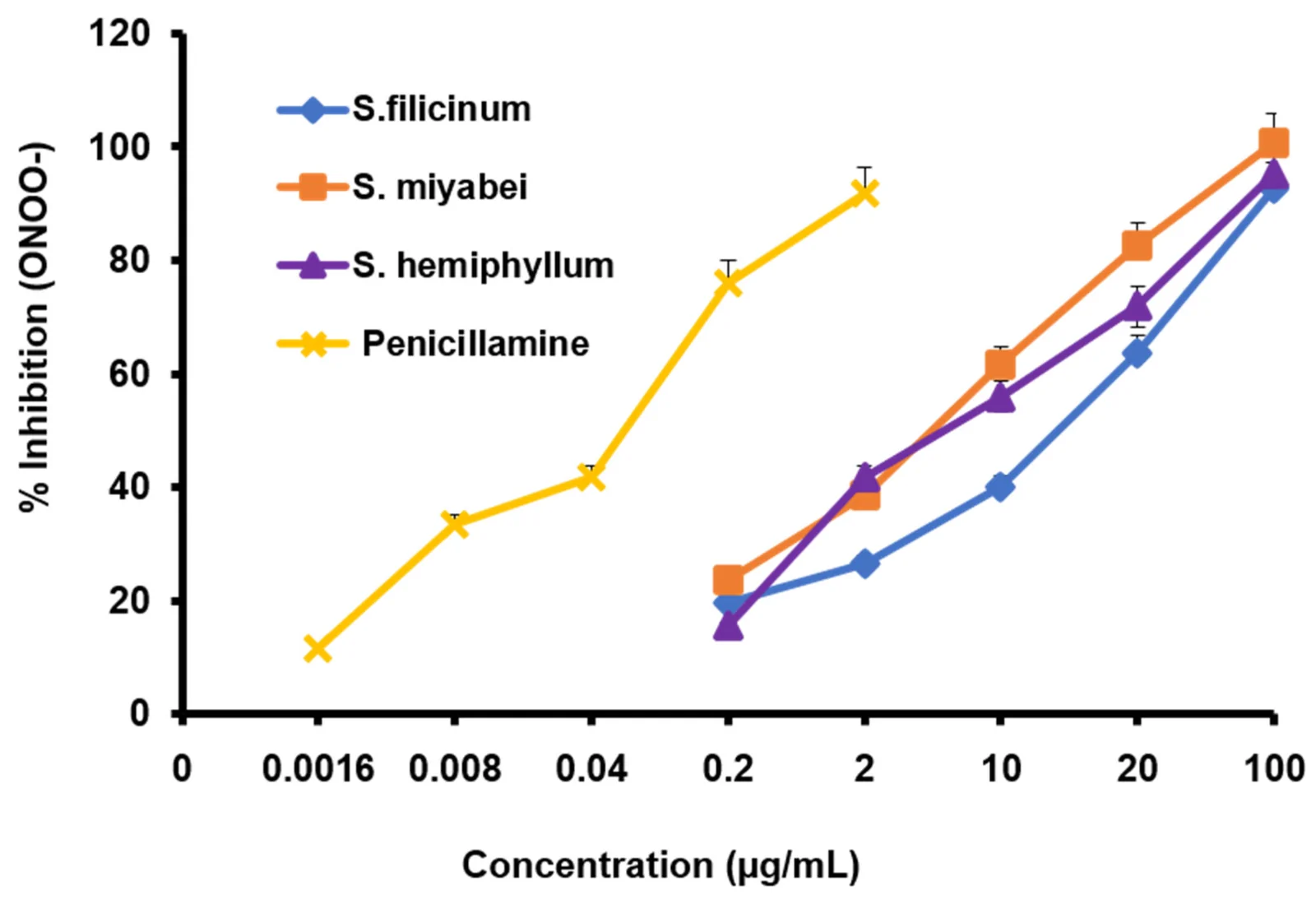
Peroxynitrite (ONOO^–^) scavenging activity of 70% EtOH *Sargassum* species extract. Penicillamine was used as a positive control. All values are expressed as the mean ± SD of triplicate experiments (*n* = 3).

**Figure 4:**
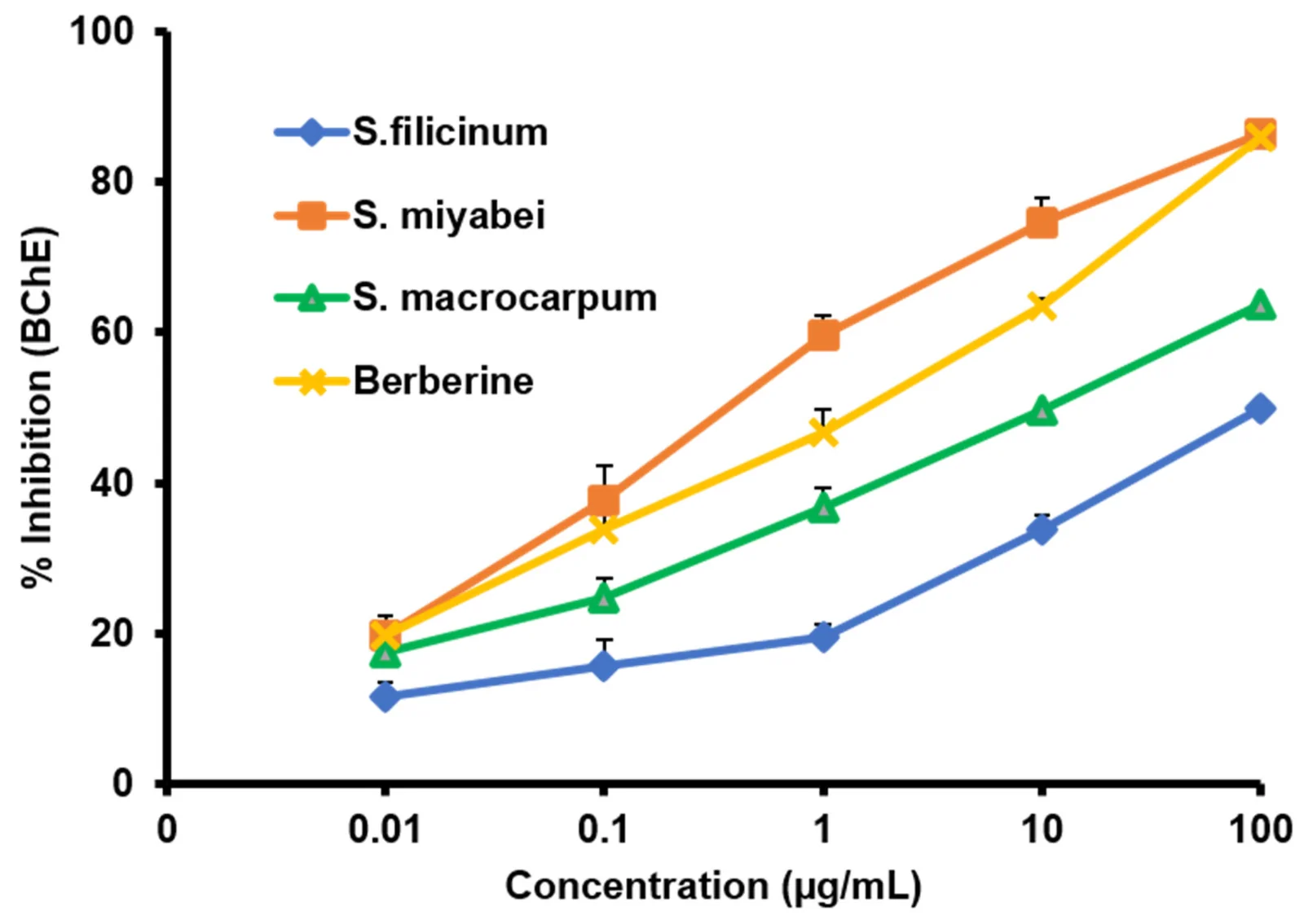
Inhibitory activity of BChE by 70% EtOH *Sargassum* species extract. Berberine was used as a positive control. All values are expressed as the mean ± SD of triplicate experiments (*n* = 3).

**Figure 5:**
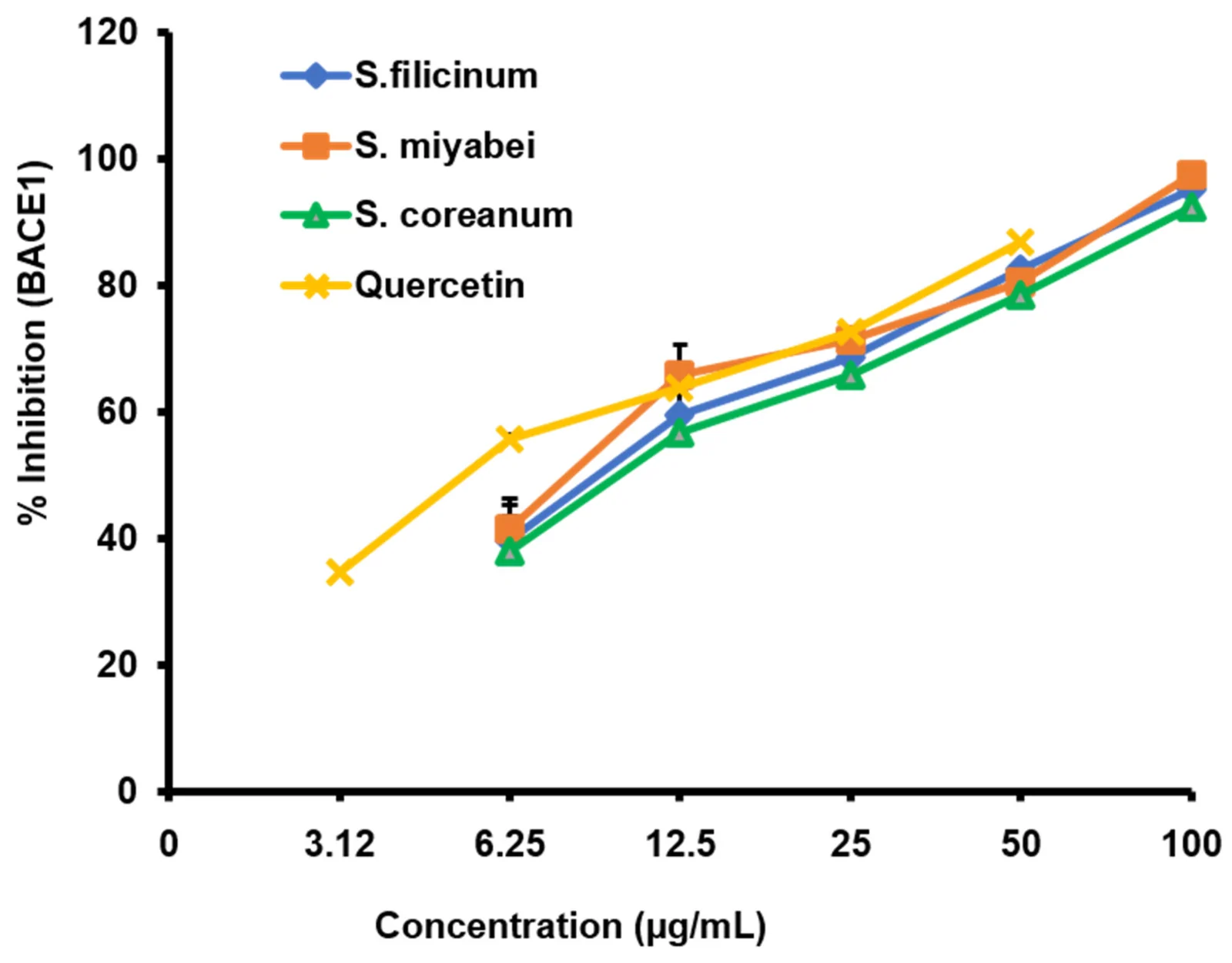
Inhibitory activity of BACE1 by 70% EtOH *Sargassum* species extract. Quercetin was used as a positive control. All values are expressed as the mean ± SD of triplicate experiments (*n* = 3).

**Figure 6:**
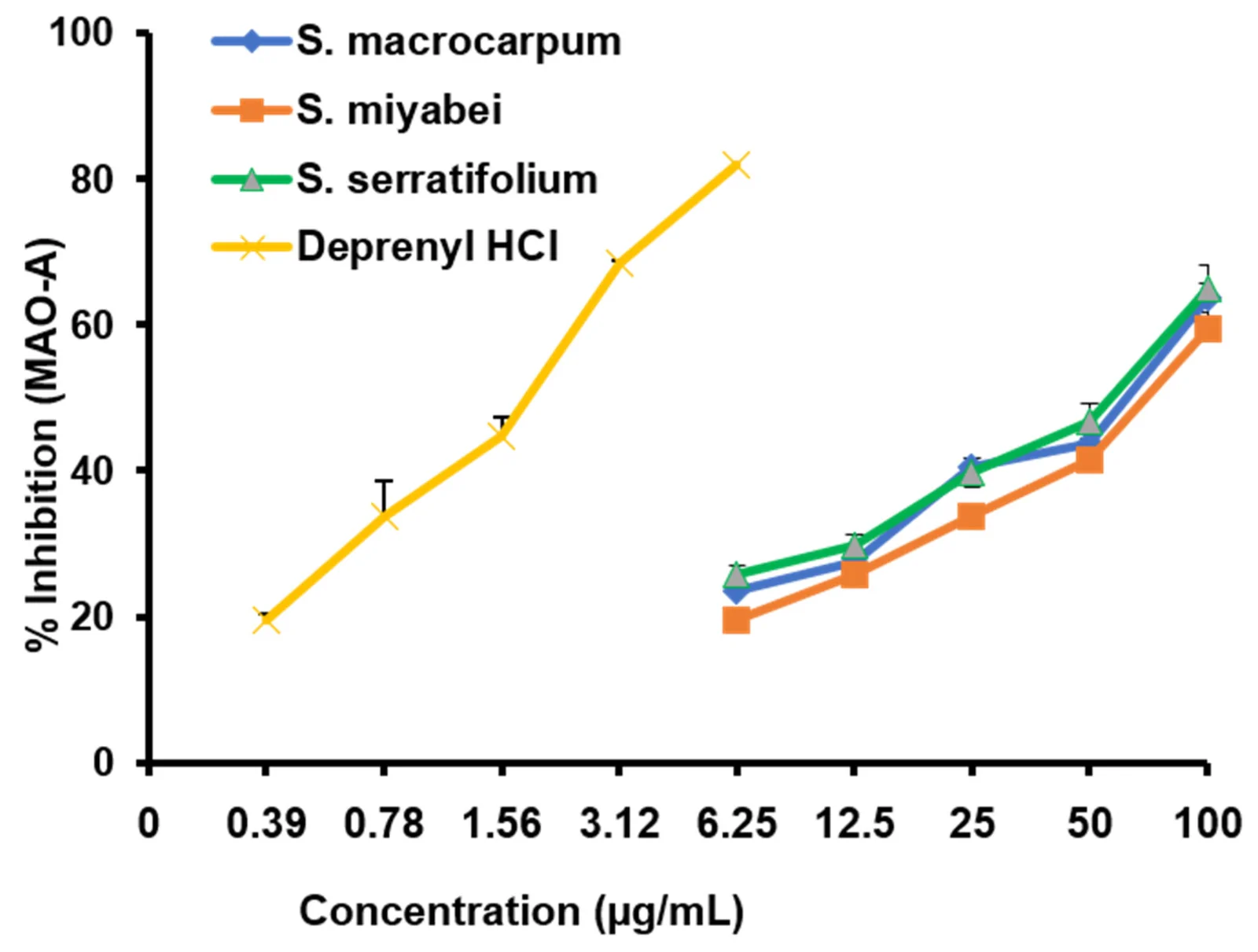
Monoamine oxidase A (MAO-A) inhibitory activity of 70% EtOH *Sargassum* species extract. Deprenyl HCl was used as a positive control. All values are expressed as the mean ± SD of triplicate experiments (*n* = 3).

**Figure 7:**
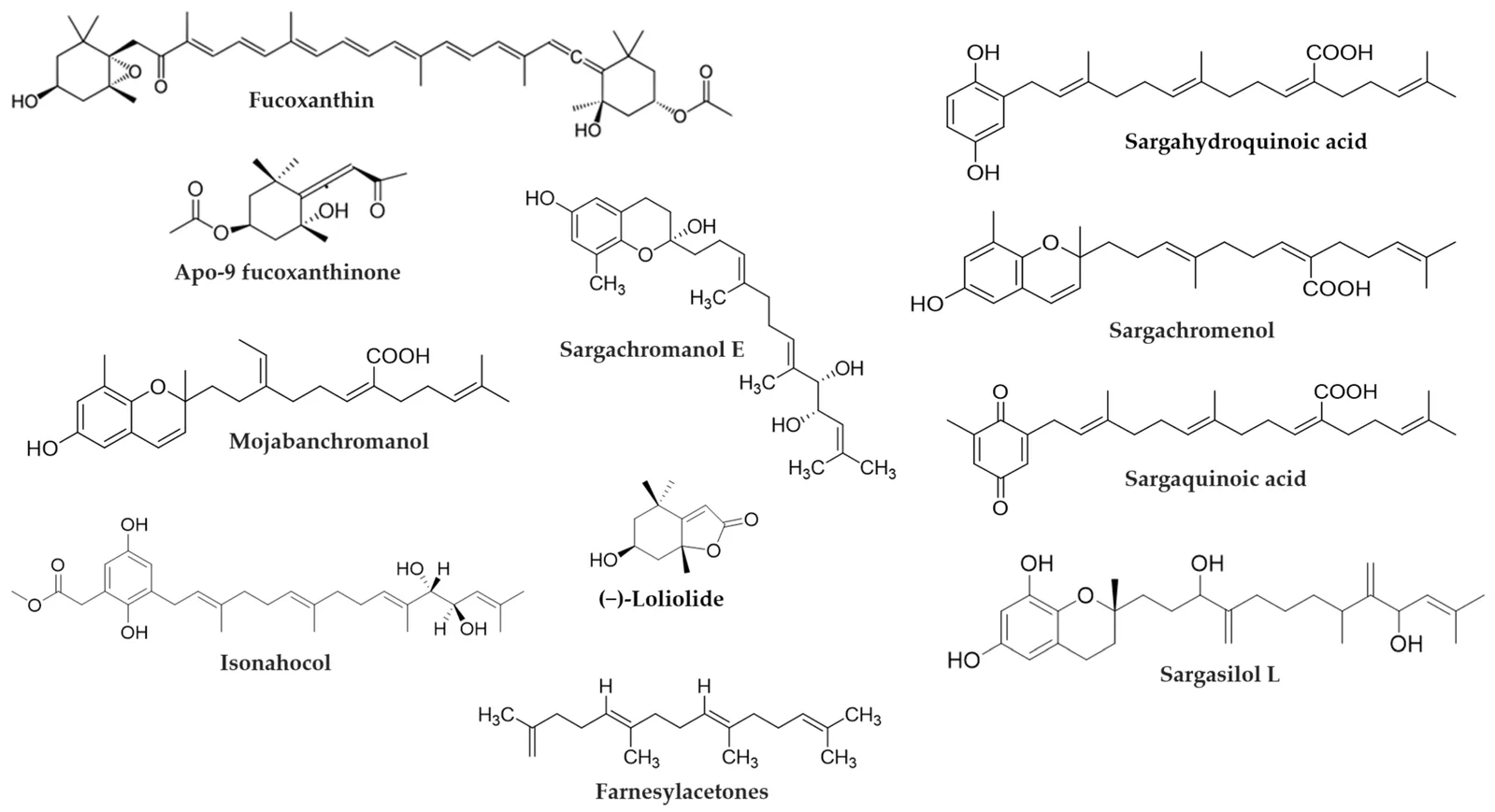
Chemical structures of major bioactive compounds previously identified in *Sargassum* species. Chemical structures were prepared using ChemDraw professional (version 20.1, PerkinElmer Informatics, Waltham, MA, USA).

**Table 1:** ONOO^–^, PTP1B, and α-glucosidase inhibitory activities of 70% ethanol extracts of various *Sargassum* and *myagroides* species (mean ± SD, *n* = 3).

Test *Sargassum* spp.	IC_50_ (μg/mL, Mean ± SD) ^a^		
	ONOO^–^	PTP1B	α-Glucosidase
*Sargassum filicinum* Harvey	4.49 ± 0.35 ***	1.87 ± 0.09 ***	2.30 ± 0.06 ***
*Sargassum siliquastrum* (Mertens ex Turner) C. Agardh	11.96 ± 0.97 **	5.71 ± 0.25 ***	51.52 ± 2.69 ***
*Sargassum miyabei* Yendo	2.46 ± 0.38 ***	6.49 ± 1.09 ***	12.12 ± 0.46 ***
*Sargassum yendoi* Okamura & Yamada	6.33 ± 0.80 ***	10.88 ± 1.53 ***	14.82 ± 0.03 ***
*Sargassum confusum* C. Agardh	15.61 ± 0.70 **	11.88 ± 0.81 ***	24.71 ± 1.28 ***
*Myagropsis myagroides* (Mertens ex Turner) Fensholt	11.28 ± 2.83 **	6.73 ± 0.84 ***	11.17 ± 0.51 ***
*Sargassum micracanthum* (Kützing) Endlicher	33.19 ± 2.89 *	40.38 ± 2.25 **	>100
*Sargassum hemiphyllum* (Turner) C. Agardh	2.07 ± 0.06 ***	2.11 ± 0.07 ***	3.67 ± 0.17 ***
*Sargassum macrocarpum* C. Agardh	17.25 ± 0.86 **	11.17 ± 0.45 ***	61.72 ± 3.21 ***
*Sargassum coreanum* J. Agardh	51.50 ± 0.35 *	15.39 ± 0.39 **	>100
*Sargassum serratifolium* (C. Agardh) C. Agardh	14.30 ± 0.63 **	10.58 ± 0.67 ***	>100
Penicillamine ^b^	0.22 ± 0.10 ***	-	-
Ursolic acid ^c^	-	7.92 ± 0.20 ***	-
Acarbose ^d^	-	-	92.97 ± 3.81 **
Control	0.00 ± 4.63	0.00 ± 5.44	0.00 ± 1.41

^a^ The 50% inhibitory concentration (IC_50_) values (μg/mL) were calculated from a log dose concentration–inhibition curve and are expressed as mean ± SD of triplicate experiments. ^b,c,d^ Used as positive controls in each assay. * *p* < 0.05, ** *p* < 0.001, and *** *p* < 0.0001 indicate significance differences from control.

**Table 2:** AChE, BChE, BACE1, MAO-A, and MAO-B inhibitory activities of 70% ethanol extracts of various *Sargassum* and *myagroides* species (mean ± SD, *n* = 3).

Test *Sargassum* spp.	IC_50_ (μg/mL, Mean ± SD) ^a^				
	AChE	BChE	BACE1	MAO-A	MAO-B
*Sargassum filicinum* Harvey	>100	93.96 ± 4.51 *	8.76 ± 0.72 ***	91.32 ± 3.99 *	>100
*Sargassum siliquastrum* (Mertens ex Turner) C. Agardh	>100	>100	10.70 ± 1.51 ***	>100	>100
*Sargassum miyabei* Yendo	>100	39.76 ± 5.29 **	3.88 ± 0.75 ***	16.91 ± 0.87 **	>100
*Sargassum yendoi* Okamura & Yamada	>100	>100	17.71 ± 2.00 **	>100	>100
*Sargassum confusum* C. Agardh	>100	>100	13.37 ± 2.80 ***	>100	>100
*Myagropsis myagroides* (Mertens ex Turner) Fensholt	>100	>100	9.74 ± 1.95 ***	>100	>100
*Sargassum micracanthum* (Kützing) Endlicher	>100	>100	63.50 ± 1.12 *	>100	>100
*Sargassum hemiphyllum* (Turner) C. Agardh	>100	>100	16.88 ± 0.49 **	>100	>100
*Sargassum macrocarpum* C. Agardh	>100	77.98 ± 3.69 *	13.14 ± 2.68 ***	14.02 ± 0.22 **	>100
*Sargassum coreanum* J. Agardh	>100	>100	7.14 ± 0.51 ***	>100	>100
*Sargassum serratifolium* (C. Agardh)	>100	>100	11.55 ± 1.49 ***	12.46 ± 0.73 **	>100
Berberine ^b^	0.34 ± 0.02 ***	11.49 ± 0.90 ***	–	–	–
Quercetin ^c^	–	–	3.5 ± 0.24 ***	–	–
Deprenyl HCl ^d^	–	–	–	2.47 ± 0.11 ***	0.31 ± 0.01 ***
Control	0.00 ± 1.62	0.00 ± 5.12	0.00 ± 3.71	0.00 ± 4.77	0.00 ± 5.14

^a^ The 50% inhibitory concentration (IC_50_) values (μg/mL) were calculated from a log dose concentration–inhibition curve and expressed as mean ± SD of triplicate experiments (*n* = 3). ^b,c,d^ Used as positive controls in each assay. * *p* < 0.05, ** *p* < 0.001, and *** *p* < 0.0001 indicate significance differences from the control.

## Data Availability

The original contributions presented in this study are included in the article. Further inquiries can be directed to the corresponding authors.
